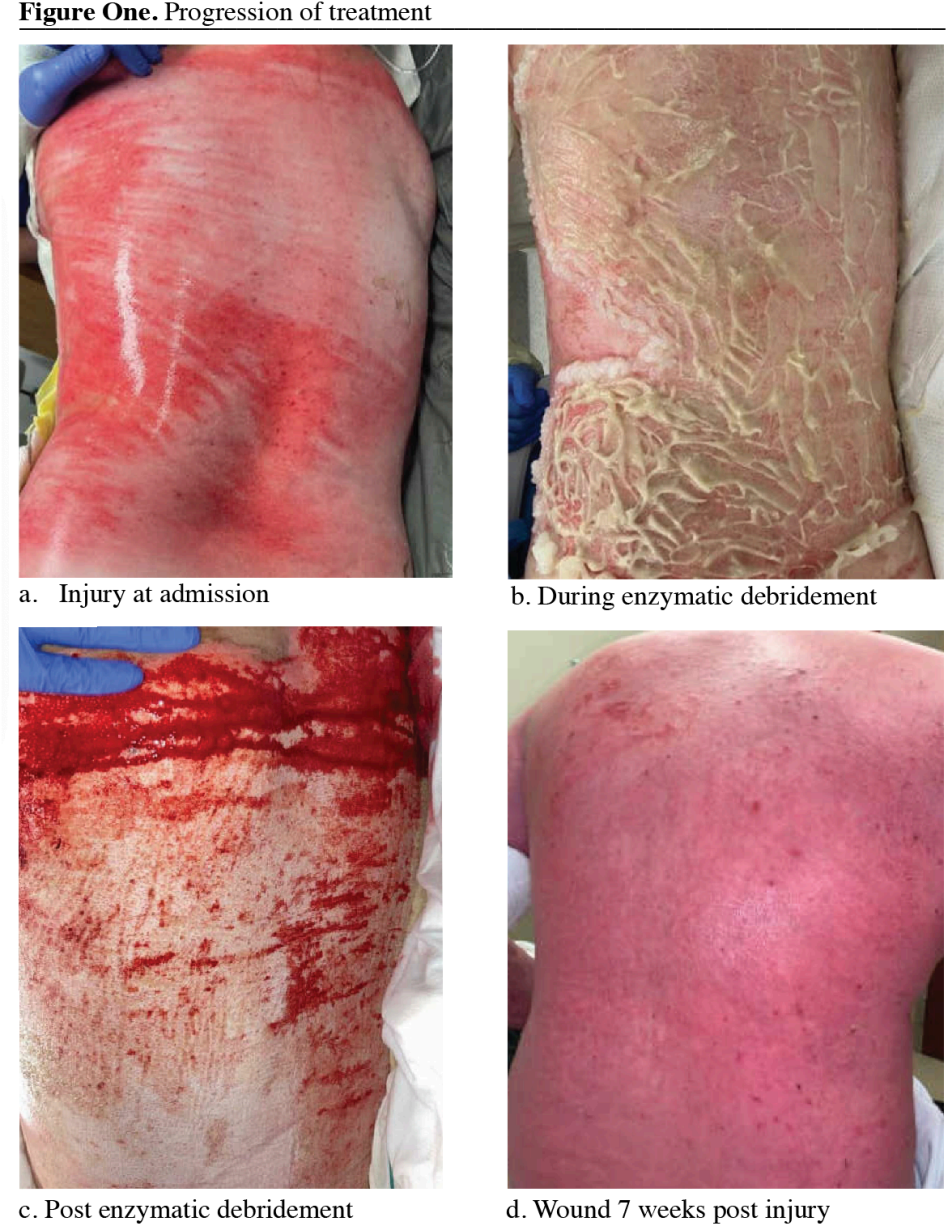# 838 Innovative Management of Pediatric Burns: Combining Enzymatic Debridement and Autologous Skin Cell Suspension

**DOI:** 10.1093/jbcr/iraf019.369

**Published:** 2025-04-01

**Authors:** Cole Bird, Dhaval Bhavsar

**Affiliations:** University of Kansas School of Medicine; The University of Kansas Health System Burnett Burn Center

## Abstract

**Introduction:**

Enzymatic debriding agents (EDA) have demonstrated potential for rapid non-surgical eschar removal. Autologous skin cell suspension (ASCS) has been shown to enhance burn wound healing. Despite their individual benefits, the combined use of EDA and ASCS in burn treatment remains under-explored in the current literature.

**Methods:**

We present a case involving a 17-year-old patient with a 46% total body surface area (TBSA) burn. We treated deep partial thickness burn injury over his entire back with EDA. This was performed in operating room while doing excision and grafting for his upper extremities. Post enzymatic debridement wound was dressed with silver impregnated foam and observed for signs of re-epithelialization. After one-week, no signs of re-epithelialization were present, so the patient was treated with ASCS.

**Results:**

The enzymatic debriding agent (EDA) achieved complete debridement. Despite initial treatment, re-epithelialization was not observed after one week. Subsequent administration of autologous skin cell suspension (ASCS) resulted in rapid healing, with full wound closure achieved within 10 days, at 3 weeks after injury.

**Conclusions:**

We present the combination of enzymatic debridement and autologous skin cell suspension for effective treatment of large surface area, deep partial burns in a pediatric patient. We were able to achieve wound healing within 3 weeks with minimal surgical intervention and preservation normal uninjured dermis as a result.

**Applicability of Research to Practice:**

This case report provides a clinical model for treating burn injuries with a combination of enzymatic deriding agents and autologous skin cell suspension.

**Funding for the Study:**

N/A